# Spatial Distribution of Oxygen Chemical Potential under Potential Gradients and Theoretical Maximum Power Density with 8YSZ Electrolyte

**DOI:** 10.1038/srep18804

**Published:** 2016-01-04

**Authors:** Dae-Kwang Lim, Ha-Ni Im, Sun-Ju Song

**Affiliations:** 1 Department of Materials Science and Engineering, Chonnam National University, 77 Yongbong-ro, Buk-gu, Gwangju, 500-757, Korea

## Abstract

The maximum power density of SOFC with 8YSZ electrolyte as the function of thickness was calculated by integrating partial conductivities of charge carriers under various DC bias conditions at a fixed oxygen chemical potential gradient at both sides of the electrolyte. The partial conductivities were successfully taken using the Hebb-Wagner polarization method as a function of temperature and oxygen partial pressure, and the spatial distribution of oxygen partial pressure across the electrolyte was calculated based on Choudhury and Patterson’s model by considering zero electrode polarization. At positive voltage conditions corresponding to SOFC and SOEC, the high conductivity region was expanded, but at negative cell voltage condition, the low conductivity region near n-type to p-type transition was expanded. In addition, the maximum power density calculated from the current-voltage characteristic showed approximately 5.76 W/cm^2^ at 700 ^o^C with 10 μm thick-8YSZ, while the oxygen partial pressure of the cathode and anode sides maintained ≈0.21 and 10^−22^ atm.

Solid Oxide Fuel Cell (SOFC) is a highly efficient electrochemical solid state device which converts chemical energy directly into electrical energy and into a valuable hot stream due to its operation at higher temperatures^1^, leading to several distinct advantages over other types of fuel cells (e.g. non-precious materials and solid electrolyte); hence, the problems of corrosion and electrolyte management are overcome^2^,^3^,^4^. SOFC has been a leading candidate for a wide range of applications in stationary power generation, transport, and portable devices^5^,^6^,^7^. The best application of SOFC systems might be stationary power generation. Depending on the size of the SOFC generator, stationary power generation can be further grouped into residential, industrial, and central markets by using various fuels^8^,^9^.

Although many applications of SOFC are possible, commercialization of SOFC on a large scale is not yet successful. This might be due to its higher operating temperature and relatively low power density. To overcome the barrier to the commercialization of SOFC technology, many researchers have tried to achieve power density by increasing the size of the cell components^3^,^4^,^5^,^10^,^11^. However, SOFC contains all ceramic components that have a brittle nature. By increasing the size of the SOFC single cell, the mechanical strength of the ceramic components can also become a critical problem. It may also increase the number of cracks and defects in the single cell of SOFC^12^,^13^,^14^. Therefore, rather than increase the size of the electrolyte, developing the best electrode materials and their microstructures and decreasing the thickness of the electrolyte to the optimum level can help to increase the power density of SOFC while maintaining mechanical safety.

To demonstrate this rational, in the present study, we report the maximum power density of SOFC that can be obtained from a given electrolyte thickness by integrating partial conductivities of charge carriers under various DC bias conditions at a fixed oxygen chemical potential gradient at both sides of the electrolyte. The partial conductivities were successfully taken from the Hebb-Wagner polarization method as a function of temperature and oxygen partial pressure (details are given in the supplement), and the spatial distribution of oxygen partial pressure across the electrolyte was calculated based on Choudhury and Patterson’s work^15^,^16^ by considering zero electrode polarization.

## Methods

### Theory of Calculations

The non-linear current-voltage characteristics of Mixed Ionic Electronic Conductors (MIEC) under an oxygen partial pressure gradient were reported to extract the partial conductivity^17^ and interfacial oxygen potential^18^,^19^. Even though the theory itself appears to be flawless, it was extremely difficult to realize experimentally so that the partial conductivities corresponding to blocking electrode conditions were only limitedly reported^20^,^21^,^22^,^23^. By extending Wagner’s ambipolar diffusion, Choudhury and Patterson^15^,^16^ begins with the parameter, *r*, the ratio of the ionic current density to the electronic current density is as follows,


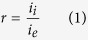


where 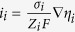 and 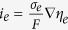. The total external current, *I*_*ext*_, and the terminal voltage, *V*_*T*_ were developed as shown below


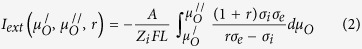



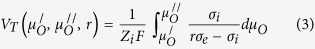


where F is Faradays constant, A is the cross-sectional area of the MIEC, L is the length of the MIEC, z_i_ is the charge state of oxygen ion, σ_k_ is the partial conductivity of k species, and (/) and (//) are superscripts referring to conditions on either side of the MIEC. By integrating Eq. 4 up to μ_O_(x) based on no interfacial polarization, the spatial distribution of the oxygen chemical potential may be obtained as


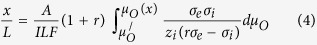


From the current-voltage characteristic, one can calculate the partial conductivity as a function of oxygen partial pressure.

On the other hand, because the partial conductivities of 8YSZ were obtained by Hebb-Wagner polarization method as shown in figure S5 of the Supplementary Information, one can successfully calculate the spatial distribution of oxygen chemical potential across material under a fixed oxygen potential gradient using Eq. 4.

## Results

A solid electrolyte, 8YSZ with 8 mol % yttria Yttria Stabilized Zirconia is chosen in this work because it is one of the most important oxygen ion conducting solid electrolytes used in the SOFC^24^,^25^, due to the high ionic conductivity and good chemical stability over a wide range of temperature and oxygen partial pressure^17^,^26^. Even though 8YSZ is a well-known electrolyte under experimental conditions, different and non-linear oxygen partial pressure distribution can cause a difference in local oxygen partial pressure gradient as a driving force for ions, leading to several series resistances to the charge carriers within the electrolyte. Due to the p-type to n-type transition in minor charge carriers, an internal leakage current should still be accounted for to achieve precise expectation of maximum power density. The successful electron and electronic defect partial conductivity measurements as functions of temperature and wide range of oxygen partial pressure by Hebb-Wagner polarization measurement (details are given in supplement) might provide the precise calculation of theoretical maximum power density with 8YSZ.

**Figure 1:**
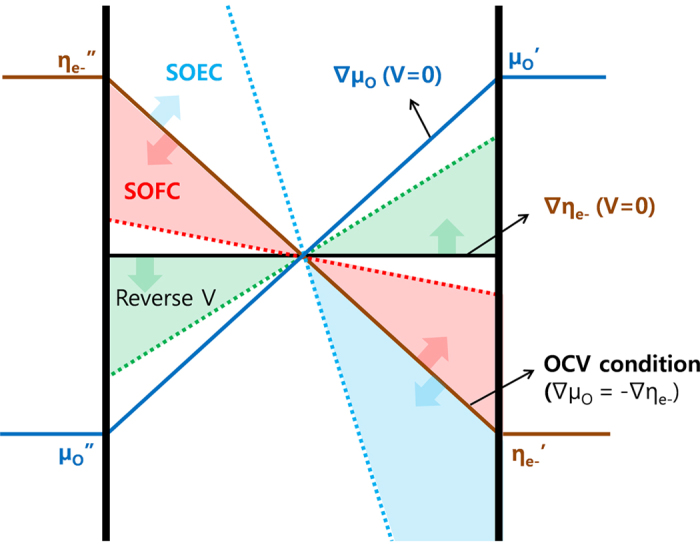
Schematic view of chemical and electrical potential applied to YSZ electrolyte.

A schematic view of cell operation behavior for various terminal voltages under a fixed oxygen partial pressure at both boundaries is shown in Fig. 1. Without considering the spatial distribution of potential within the electrolyte, the linear chemical potential profile and DC bias may be assumed conceptually to show the direction of charge carrier movement forced by external forces. At SOFC operation conditions, the high and low oxygen partial pressure at both electrodes could be around 0.21 and 10^−22^ atm similar to the fuel cell operating condition, respectively.

At open cell voltage (OCV) condition, the electrochemical potential gradient of electron across the electrolyte can be given in the opposite direction against the chemical potential gradient from ∆G = −nEF and V = −∇η_e_/e, and ionic flows from the chemical potential gradient and electrochemical potential gradient as a driving force should be equal due to the balance between the chemical and electrical forces on the ions. Where terminal voltage, V, is higher than OCV, the ionic flow due to the electric voltage applied becomes larger than that of the chemical potential gradient, leading to the SOEC mode. On the other hand, where the terminal voltage, V, is lower than OCV with a positive value, the ionic flow driven by the chemical potential gradient through the electrolyte becomes dominant, leading to the SOFC mode. It is also interesting that the regime with negative terminal voltage, which often appears as a failure of the SOFC stack operation, is caused by the higher resistant damaged cell within a stack.

**Figure 2:**
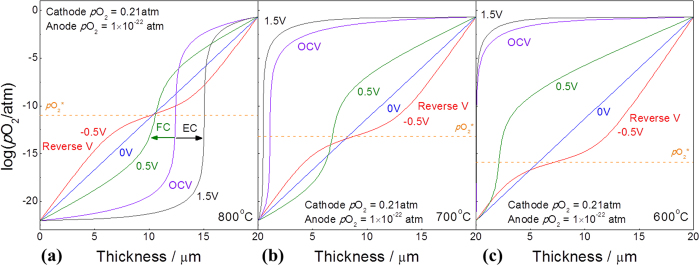
Spatial distribution of oxygen chemical potential across 8YSZ under a fixed oxygen potential gradient with DC bias.

As reported by Choudhury *et al.*^16^, the spatial distribution of oxygen partial pressure within the electrolyte may be calculated using Eq. 4 if the partial conductivities of ions, electrons, and holes are known as a function of oxygen partial pressure and temperature. The spatial oxygen chemical potential profiles across 8YSZ under a fixed oxygen partial pressure gradient with various DC biases were successfully calculated as shown in Fig. 2. As shown, the oxygen partial pressure distribution can be strongly affected by terminal voltage. Depending on the SOFC (0 < V < Vocv), SOEC (V > Vocv), and negative cell voltage (V < 0) mode, the spatial distribution of oxygen partial pressure can be distinguished from each other, leading to the different average electrical properties.

Chemical potential distribution across the 8YSZ electrolyte may be qualitatively understood while various terminal voltages are applied.


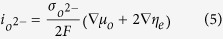



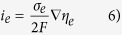


Where forward bias was applied (terminal voltage V > 0), the electrochemical potential of the electron, η_e_, should be applied in the opposite direction than that of ∇μ_o_, and ∇η_e_ should be maximum at the n-p transition point, which is the highest resistivity region. At a steady state condition, the total current flux should be constant so that ∇μ_o_ reaches maximum where ∇η_e_ is maximum because the oxide ion flux driven by the electric force should flow in the opposite direction from that of chemical force. As a result, the maximum conductivity regime can be extended and the minimum conductivity regime can be minimized as shown in Fig. 2(a–c). By integrating partial conductivity across the specimen, one can extract the average electrical property at a given condition and it is shown that as greater forward bias is applied, higher conductivity can be obtained because the high resistivity n-p transition region can be smaller. As the temperature decreases, the n-p transition point can shift to the lower oxygen partial pressure and maximum conductivity region on the high oxygen partial pressure side becomes overwhelming, even though the difference in conductivity should be small enough for all the specimens to be under the electrolyte regime at given thermodynamic conditions.

When the terminal voltage becomes greater than OCV, the highest conductivity region expands and the n-type to p-type transition region moves to a lower oxygen partial pressure side at 800 ^o^C, to a higher oxygen partial pressure side at 700, and 600 ^o^C, respectively. From the results, one can expect that the overall oxygen potential of the electrolyte can transit from an under reducing to an oxidizing condition as temperature decreases at the SOEC mode.

At negative cell voltage conditions, the electrical potential field at the n-type to p-type transition region may be larger than that around both sides and the chemical potential around the minimum conductivity region becomes dominant, showing minimum conductance of the electrolyte. This can be explained by the fact that where backward bias was applied (terminal voltage V < 0), the electrochemical potential of the electron should be applied in the same direction as that of ∇μ_o_. At the steady state condition, the total current flux should be constant so that ∇μ_o_ reaches minimum where ∇η_e_ is maximum because the oxide ion flux driven by the electric force should flow in the same direction as that of the chemical force.

## Discussion

**Figure 3:**
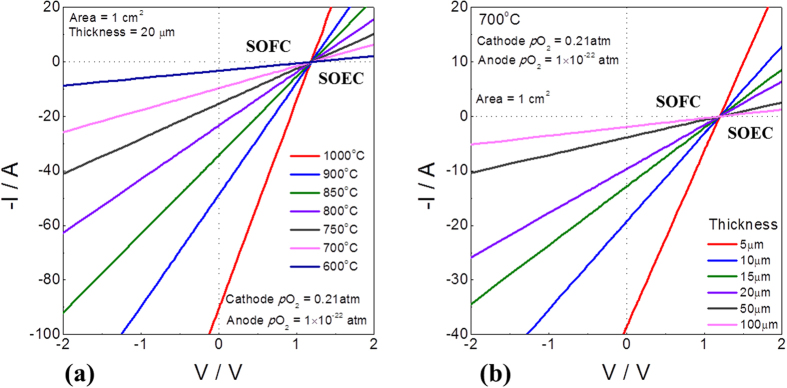
Current-voltage characteristic of YSZ electrolyte under chemical potential gradient (at high oxygen partial pressure = 0.21 atm, at low oxygen partial pressure 10^−22^ atm).

The theoretical maximum power density with the 8YSZ electrolyte can be used as an evaluating tool for the development of electrodes materials. In many cases, power densities of SOFC were reported with novel electrode materials or microstructure, but no comparison with a theoretical limit was given so that one cannot be sure the degree of upgrade of the cell might still be possible. Based on both the partial conductivities as a function of oxygen partial pressure and oxygen partial pressure distribution within the electrolyte with terminal voltage, the theoretical maximum power density can be precisely calculated as given in Fig. 3. The current-voltage characteristics at fuel cell conditions with temperature and thickness dependence are given in Figs 3(a,b), respectively. The typical linear relationship for both cases was observed because of the electrolytic properties of 8YSZ. At 700 ^o^C with 10 μm thick YSZ, the theoretical maximum power density was around 5.76 W/cm^2^, as given in Table 1. Note that the oxygen partial pressure of the cathode and anode sides was 0.21 and 10^−22^ atm, respectively. Even the thick electrolyte cell with 50 μm 8YSZ might provide the maximum power density of 1.15 W/cm^2^ at 700 °C, implying that efforts should be made to develop electrode optimization to achieve better cell performance, rather than to enlarge the cell size possibly.

## Conclusion

The spatial distribution of oxygen partial pressure across the YSZ in an oxygen chemical potential gradient was successfully calculated as a function of various terminal voltages, based on the partial conductivities measured from Hebb-Wagner polarization measurements. At positive voltage conditions, corresponding to SOFC and SOEC, the high conductivity region was expanded, but at negative cell voltage condition, the low conductivity region near the n-type to p-type transition was expanded. In addition, the maximum power density calculated from the current-voltage characteristic is around 5.76 W/cm^2^ at 700 ^o^C, while the oxygen partial pressure of the cathode and anode sides maintains ≈0.21 and 10^−22^ atm.

## Additional Information

**How to cite this article**: Lim, D.-K. *et al.* Spatial Distribution of Oxygen Chemical Potential under Potential Gradients and Theoretical Maximum Power Density with 8YSZ Electrolyte. *Sci. Rep.* **6**, 18804; doi: 10.1038/srep18804 (2016).

## Supplementary Material

Supplementary Information

## Figures and Tables

**Table 1:** Theoretical maximum power density with 8YSZ under chemical potential gradient (at high oxygen partial pressure = 0.21 atm, at low oxygen partial pressure 10^−22^ atm).

Temp.	Max. Power density (Wcm^−2^) (Thickness = 20μm)	Thickness	Max. Power density (Wcm^−2^) (Temp. = 700 °C)
1000 °C	26.84	5μm	11.53
900 °C	14.48	10μm	5.76
850 °C	10.22	15μm	3.84
800 °C	6.96	20μm	2.88
750 °C	4.56	50μm	1.15
700 °C	2.88	80μm	0.72
600 °C	0.94	100μm	0.57
