# Supplementary material for: Spatial Distribution of Oxygen Chemical Potential under Potential Gradients and Theoretical Maximum Power Density with 8YSZ Electrolyte
Source: Sci Rep. 2016 Jan 4;6:18804. doi: 10.1038/srep18804 (PMC4698742; doi:10.1038/srep18804)
Supplement: Supplementary Information [file srep18804-s1.pdf]

Supplementary Information

Spatial Distribution of Oxygen Chemical Potential under Potential Gradients and Theoretical Maximum Power Density with 8YSZ Electrolyte

Dae-Kwang Lim, Ha-Ni Im, Sun-Ju Song

Department of Materials Science and Engineering, Chonnam National University,

300 Yongbong-dong, Buk-gu, Gwangju, 500-757, Korea

Corresponding author: Prof. Sun-Ju Song

Tel: +82-62-530-1706

Fax: +82-62-530-1699

E-mail: song@chonnam.ac.kr

An electrolyte needs to have high ionic and low electric conductivities. Electric conductivity is normally insensitive to chemical potential, and maintains its conductive characteristics at the presence of a chemical potential gradient. However, determining its electrolytic domain by conductivity measurement is an important subject for the operation of a solid electrolyte fuel cell. YSZ, an excellent ionic conductor, shows a very low electric conductivity, and researchers have thus paid less attention to this issue. Note, however, that electric conduction can limit the cell efficiency by reducing the driving force.

In this study, we measured electric conductivity using the Hebb-Wagner polarization method[1]. The method uses an ion-blocking electrode, and measures the genuine electric conduction quantitatively by generating a sufficiently large chemical potential difference to offset the applied external voltage difference to drive transport[2,3].

Experimental

A commercial powder (8YSZ, Totho) with an average particle size of 40 nm was formed into a disc in CIP under 150 MPa, and sintered at 1450℃ for 10 hr in air. We cut the sintered polycrystalline 8YSZ specimen into a 1.5 mm thick disc for the Hebb-Wagner polarization experiment. We also cut 1.8x1.8x11 mm samples from the specimen and polished their surfaces to a roughness of less than 1 μm for conductivity measurement. Supplementary Figure S1 shows the X-ray diffraction pattern of 8YSZ, scanned at 2°/min by XRD (D/MAX Ultima III, Rigaku, Japan) and analyzed the pattern by profile matching.

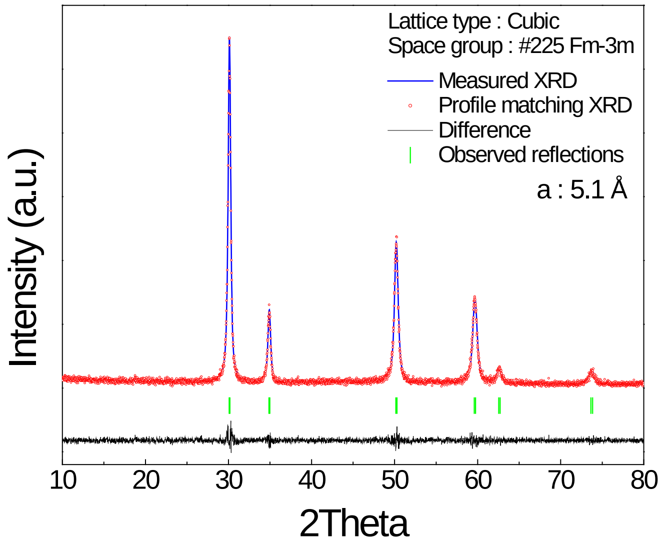

Supplementary Figure S1. Room-temperature XRD pattern of the 8YSZ.

Supplementary Figure S2 shows an image of fractured 8YSZ using scanning electron microscopy (SEM, SS-550, Shimadzu, Japan), which indicates the very dense nature of the specimen. Its bulk density measured using the Archimedes method was higher than 97.5%.

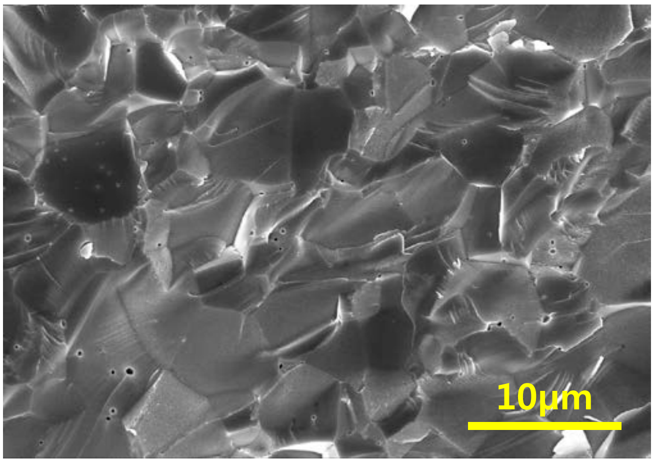

Supplementary Figure S2. SEM image of the fractured 8YSZ sintered at 1450℃ in air.

We measured the electric conductivity of samples using the 4-probe method, combining a digital multimeter (Keithley 2700) and a current source (Keithley 6220) at various temperatures (700 ≤ Temp. (℃) ≤ 1000) and partial pressures of oxygen (0.21 ≤ pO_2 (atm.) ≤ 10^{-23}). The partial conductivity of 8YSZ was measured by preparing a Hebb-Wagner polarization cell with an ion-blocking electrode (Pt foil) on one side and a reversible electrode (Pt mesh) on the other.

Results and Discussion

Configuration of Hebb-Wagner polarization cell

The four-probe setup was shown in Supplementary Figure S3 to eliminate the possibility of the underestimation of electric conductivity, which often occur with two electrodes due to over-voltage at the electrodes. Mass and charge transport through the area other than that of the electrode was completely blocked off by double sealing.

$$\mathrm{p_{O_2}^{I},\, RE|YSZ|p_{O_2}^{II},\, IBE(Pt\,foil)}$$

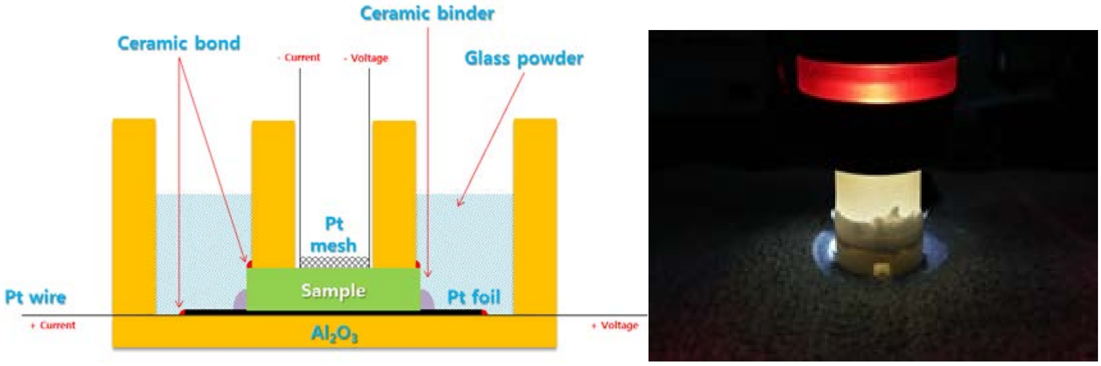

Supplementary Figure S3. Schematic and photo of the Hebb-Wagner polarization cell in a four-probe setup.

Partial conductivity from Hebb-Wagner polarization

Thus, by measuring the voltage of both ends of the sample at steady-state, the oxygen chemical potential difference can be estimated. Supplementary Figure S4 shows the profile of the current-voltage characteristic of the 8YSZ, reaching the steady-state. Since current density at steady-state is due only to the electron, the slope of the current-voltage curve can provide partial electric conductivity according to the following equation:

$$\sigma_e\left(\mu_{O_2} = \mu_{O_2}^{//}\right) = -\frac{L}{A}\left(\frac{dj_e}{dU}\right) \tag{S-1}$$

Here, L, A, and U represent the length, area, and voltage at both ends of electrodes in the sample, respectively.

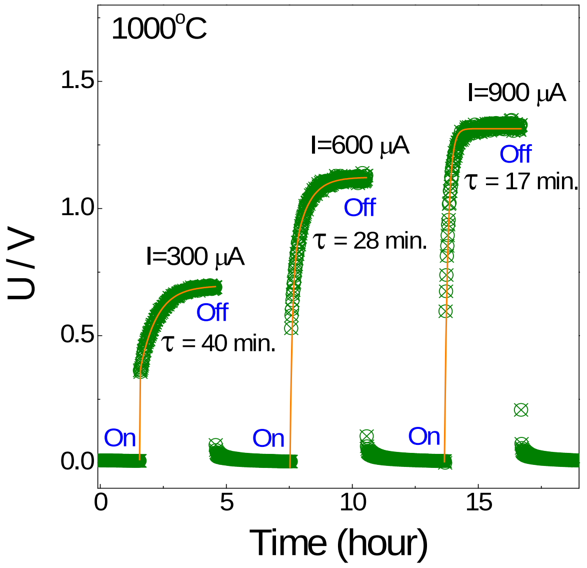

Supplementary Figure S4. Current-voltage characteristic of the 8YSZ.

It is apparent that the curves of the OCV state at the applied constant current consist of a drastic increase in voltage, followed by convergence to the steady state. It is also confirmed that, if applied constant current is shut off, the curves revert to the original state with no voltage difference.

Electric conductivity is composed of contributions from the electron and hole:

$$\sigma_e = \sigma_n + \sigma_p \tag{S-2}$$

In the case of YSZ at the given partial pressures of oxygen (0.21-10^{-23} atm.), the conductivities by electron and hole are proportional to the partial pressure of oxygen to the powers of –1/4 and 1/4, respectively:

$$\sigma_e = \sigma_n^* \left(\frac{pO_2}{pO_2^{\ *}} \right)^{-1/4} + \sigma_p^* \left(\frac{pO_2}{pO_2^{\ *}} \right)^{1/4} \tag{S-3}$$

Therefore, considering the chemical potential of the oxygen molecule and the partial pressure of oxygen, the partial conductivities of the electron and hole under the applied constant current and voltage at steady-state is:

$$I_e = \frac{ART}{LF} \left[\sigma_n^* \left\{ \exp\left(\frac{EF}{RT} \right) - 1 \right\} + \sigma_p^* \left\{ 1 - \exp\left(-\frac{EF}{RT} \right) \right\} \right] \tag{S-4}$$

The above equation indicates that the current is a function of electron and hole conductivities at the specific partial pressure of oxygen. Therefore, voltage measurement at the steady-state under a specific partial pressure of oxygen at applied constant current can provide data sets as shown in Supplementary Figure S5.

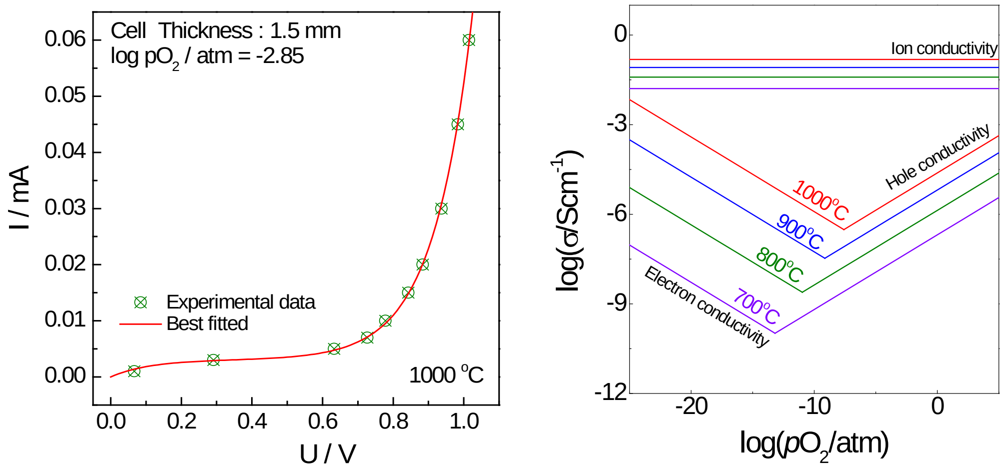

Supplementary Figure S5. Partial conductivities of the 8YSZ.

Based on these data after proper fitting, we calculated the partial conductivity of the electron and hole in the 8YSZ system as:

$$\sigma_n = \left(2.42 \pm 0.23 \times 10^7\right)\exp\left(-3.99 \pm 0.17 eV / kT\right)\left(pO_2\right)^{-1/4} \quad \text{(S-5)}$$

$$\sigma_p = \left(1.30 \pm 0.15 \times 10^2\right)\exp\left(-1.70 \pm 0.06 eV / kT\right)\left(pO_2\right)^{1/4} \quad \text{(S-6)}$$

In addition, by using the calculated partial electric conductivities and the total conductivity by the 4-probe DC measurement, we calculated the ionic conductivity as:

$$\sigma_{ion} = \left(2.24 \pm 0.10 \times 10^2\right)\exp\left(-0.80 \pm 0.01 eV / kT\right) \quad \text{(S-7)}$$

Under the normal partial pressure of the oxygen range (Air-H_2) in SOFC, 8YSZ showed superior ionic conductivity, and was in good agreement with the reported ionic conductivity of 0.79 eV[4] within the error range. From these results, the upper and lower limits of oxygen partial pressure (P_p and P_n) and oxygen partial pressure (P_{pn}) where concentrations of electron and hole become equal are calculated in the electrolyte domain, where the transport number of oxygen ion is above 0.5:

$$P_p = \left(\frac{A_i}{A_p}\right)^4 \exp\left[-\frac{4(E_i - E_p)}{kT}\right] \quad \text{(S-8)}$$

$$P_n = \left(\frac{A_i}{A_n}\right)^{-4} \exp\left[\frac{4(E_i - E_n)}{kT}\right] \quad \text{(S-9)}$$

$$P_{pn} = \left(\frac{A_n}{A_p}\right)^2 \exp\left[-\frac{2(E_n - E_p)}{kT}\right] \quad \text{(S-10)}$$

As shown in Supplementary Figure S6, the electrolytic domain covers wide ranges of both partial pressure of oxygen and temperature. For example, the electrolytic domain at $t_{ion} \geq 0.99$ at 700℃ extends the oxygen partial pressure range across 10^{11}-10^{-38} atm., while it is 4.5 atm. at 2000℃, which is in good agreement with the reported values by Park et al[4].

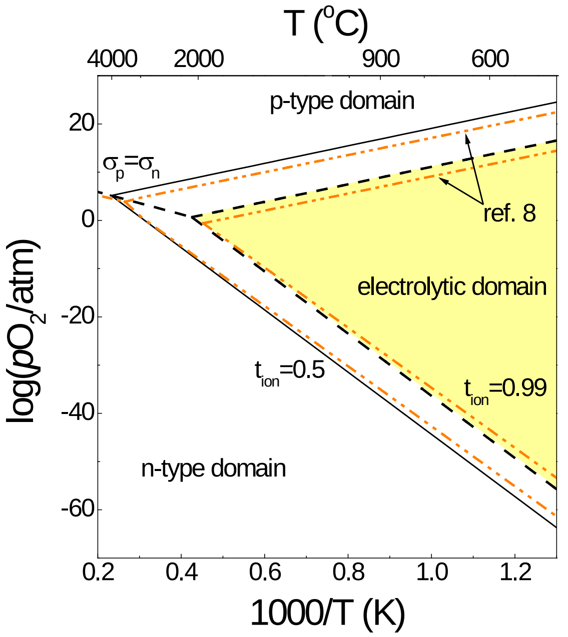

Supplementary Figure S6. Calculated boundaries in electrolytic domain of the 8YSZ.

Reference

1. Lim, D.-K., Guk, J.-G., Choi, H.-S. & Song, S.-J. Measurement of partial conductivity of 8YSZ by Hebb-Wagner polarization method. *J. Korean Ceram. Soc.*, **52(5)**, 299-303 (2015).

2. Hebb, M. H. Electrical Conductivity of Silver Sulfide. *J. Chem. Phys.*, **20,** 185-190 (1952).

3. Kim, J. H. & Yoo, H. I. Partial electronic conductivity and electrolytic domain of $La_{0.9}Sr_{0.1}Ga_{0.8}Mg_{0.2}O_{3-\delta}$. *Solid State Ion.*, **140,** 105-113 (2001).

4. Park, J. H. & Blumenthal, R. N. Electronic Transport in 8 Mole Percent Y_2O_3 - ZrO_2. *J. Electrochem. Soc.*, **136(10)**, 2867-2876 (1989).
